# Influence of Roasting Temperatures on the Antioxidant Properties, β-Glucan Content, and Volatile Flavor Profiles of Shiitake Mushroom

**DOI:** 10.3390/foods10010054

**Published:** 2020-12-28

**Authors:** In-Seo Hwang, Seo-Yeong Chon, Woo-Suk Bang, Mina K. Kim

**Affiliations:** 1 Department of Food Science and Human Nutrition and Fermented Food Research Center, Jeonbuk National University, 567 Baekjedaero, Deokjin-gu, Jeonju-si 54896, Jeonbuk , Korea; his0753@naver.com (I.-S.H.); tjdud1128@naver.com (S.-Y.C.); 2 Department of Food and Nutrition, Yeungnam University, 280, Daehak-ro, Gyeongsan 712-749, Korea

**Keywords:** shiitake mushroom, beta-glucan, antioxidant properties

## Abstract

The objective of this study was to determine the influence of roasting conditions on the volatile flavor profiles and functional properties of shiitake mushrooms. Six different roasting temperatures between 80 °C and 180 °C with 20 °C increments were selected, and mushrooms were roasted for 60 min in a conventional oven. Roasting shiitake mushroom at 140 °C showed the highest levels of antioxidant activities including 2,2’-azino-bis (3-ethylbenzothiazoline-6-sulfonic acid) (ABTS) and 2,2-diphenyl-1-picrylhidrazyl (DPPH) radical scavenging activities, total phenols and polyphenol contents. The β-glucan ranged from 34.85% to 41.49%, and it was highest when the mushrooms were roasted at 120 °C, followed by 140 °C. Instrumental flavor analysis was conducted by Gas Chromatography using Purge and Trap, and identification of compounds were produced by NIST library. Twenty-six volatile flavor compounds were identified. The concentrations of pyrazines and furans increased with increased roasting temperatures. Shiitake mushrooms roasted at 160 °C for 60 min had the most diverse volatile flavor compound profiles. This study revealed how roasting temperatures can modulate antioxidant, functional (β-glucan) and flavor benefits.

## 1. Introduction

The shiitake mushroom (*Lentinus edodes*), one of the most cultivated mushroom in the world, has been widely incorporated into various cuisines, especially in East Asia [1]. Due to the presence of various bioactive compounds, including vitamins B1, B2, and C, dietary fiber, folate, niacin, polysaccharides, some minerals and ergosterols, consumers eat shiitake mushrooms for their unique taste and nutritional benefits [2]. In Korea, the consumption of shiitake mushrooms has been increasing since 2000; around 30,000 metric tons were consumed as of 2005 [3]. Commercialized cultivation is also available, by inoculation of mushroom spore in the dried wood, and cultivation is at its optimal at the temperature around 10–20 °C with 60–70% humidity. Shiitake mushrooms can be harvested from March to September in Korea. Mushrooms harvested in March are regarded as the best quality, as these give the most intense aroma with a unique texture. Consequently, many producers harvest mushrooms in March and distribute the mushrooms year round after processing (drying, freeze-drying, and/or spray drying). During the drying process, the taste and aroma components are concentrated due to the moisture loss, thus enhancing the unique taste of shiitake mushrooms [1].

Many studies related to shiitake mushrooms have been conducted, especially regarding the functional properties, the identification and extractions of the taste components, and flavor analysis. For example, β-glucan found in high levels in shiitake mushrooms has beneficial health functional properties: for example, anti-tumor and anti-cancer activities [4,5,6]. High antioxidant properties and an effect to decrease levels of blood cholesterol have also been reported [2,7,8]. Studies reporting shiitake mushrooms’ flavor characteristics can be classified into two groups: non-volatile compounds responsible for umami tastes and volatile compounds responsible for mushroom aromatics [9,10]. Non-volatile components including free amino acids, nucleotides and soluble carbohydrates are well-known compounds responsible for umami tastes; the aqueous extracts of shiitake mushrooms have therefore been widely utilized in the preparation of vegetable broths (dashi) and flavor enhancers [1,10]. 1-octen-3-ol has been well reported as the key aroma compound responsible for raw mushroom aromatics [9,11]. In addition to 1-octen-3-ol, many sulfur-containing compounds, including dimethyl disulfide (DMDS), dimethyl trisulfide (DMTS), and cyclic sulfur compounds, have been identified as key contributors to the distinctive shiitake mushroom flavor [12]. However, the above-mentioned studies focused only on the volatile flavor analysis or the functional benefits of shiitake mushrooms. A comprehensive understanding of shiitake mushrooms in relation to flavor and functionality is still missing. The objective of this study was to determine the influence of roasting conditions on the volatile flavor profiles and functional properties of shiitake mushrooms.

## 2. Materials and Methods

### 2.1. Sample Preparation

The shiitake mushrooms included in this study were purchased through direct contact with a local producer (Suhwooms, Iksan, South) and stored at 4 °C before roasting. The mushrooms were sliced at a thickness of four millimeters before roasting. The thickness and roasting conditions of the shiitake mushrooms were selected based on a PI’s preliminary study. Six roasting temperatures were selected: 80 °C, 100 °C, 120 °C, 140 °C, 160 °C, and 180 °C. All the samples were roasted for 60 min since sample roasted for 60 min showed the highest sensory acceptance in previous study. using a commercial convection oven (COR-030K, Dongyang-magic, Seoul, Korea). After roasting, samples were stored at room temperature in dried atmosphere with moisture absorber.

### 2.2. Antioxidant Properties

The polyphenolic compounds (free- and bound-) of shiitake mushrooms were extracted using the Folin-Ciocalteu method of Krygier, Sosulski, and Hogge (1982). Result from this method was expressed as mg (+)-catechin equivalents per 100 g of shiitake mushrooms [13]. First, 3 g of roasted shiitake mushrooms were finely ground using an electronic blender (NINJA BL682KR, Hai Xin Technology Company, Shenzhen, China), and 20 mL of 80% ethanol was added and thoroughly mixed using a vortex mixer (VM-10, Daihan Scientific Co., Wonju, Korea) at room temperature (25 °C) for 10 min. Then, this mixture was centrifuged at 10,000 rpm for 10 min. Upon completion, the supernatant was collected and further concentrated at 40 °C to 2 mL using a rotary vacuum evaporator. Then, 20 mL of distilled water was added and the supernatant was stored at −20 °C til further analysis. The residues separated from the supernatant solution were hydrolyzed using 4 mL of 4N NaOH for sixty minutes and adjusted to pH 2 with 6 N HCl. Then, 4 mL of ethyl acetate was added and thoroughly mixed into the solution, and the residues were collected; this procedure was repeated six times. The collected extracts were further concentrated to 2 mL by a rotary vacuum evaporator at 40 °C; distilled water was added up to 10 mL, and this extracts were stored at −20 °C until further analysis.

Gallic acid (Sigma-Aldrich, St. Louis, MO, USA) was used for the standard curve, and total polyphenol content was reported in the mg GAE/100 g sample. A mixture of 0.2 mL of the extract with 0.2 mL Folin-Cioculteu reagent was left at room temperature for three minutes. Then, 0.4 mL of 10% Na_2_CO_3_ and 4 mL of distilled water was added and left in the dark room for sixty minutes. A 1 mL of final mixture was inserted in a cuvette (Ratiolab cuvets, semi-micro, Hungary) and the absorbance was measured at 720 nm using a spectrophotometer (BioDrop, MET Laboratories, Inc., Baltimore, MD, USA). All analysis was conducted in triplicate.

The DPPH radicals of the extracts were measured using a previously reported method with minor modifications [14]: 4 mL of 0.2 mM DPPH solution and 4 mL of extract were thoroughly mixed and left in a dark room for 10 min at room temperature. The absorbance was measured at 520 nm using a spectrophotometer (BioDrop, MET Laboratories, Inc., Baltimore, MD, USA). The DPPH radical scavenging activity (%) was calculated by the Equation (1) as below:

Radical scavenging activity (%) = ((1 − A_sample_)/(A_control_)) × 100 (1)

where A_sample_ represents the sample absorbance and A_control_ represents the control absorbance.

The scavenging activity of the shiitake extract was measured using the ABTS^+^ decolorization assay method. After mixing 7 mM (Sigma Chemical Co., St. Louis, MI, USA) and 2.45 mM of potassium persulfate, the solution was left in the dark room for one day at the room temperature (25 °C) to form ABTS^+^. The solution was then diluted with distilled water to obtain an absorbance of 1.4–1.5 at 734 nm. The absorbance of a mixture of 50 μL of the extract solution and 1 mL of a diluted ABTS^+solution was measured at 734 nm using a spectrophotometer (BioDrop, MET Laboratories, Inc., Baltimore, MD, USA). Trolox (Sigma Chemical Co., St. Louis, MI, USA) was used to obtain the standard curve. The Trolox equivalent antioxidant capacity (TEAC) was calculated using Equation (2) as below:

TEAC (mg Trolox eq) = (ΔA_sample_)/(ΔA_Trolox_) × (*TC/SC*) (2)

where ΔA(sample) represents the change of absorbance when extracts are added, and ΔA(Trolox) represents the change in absorbance when Trolox standard solution is added, *TC* represents the concentration (mg/mL) of the Trolox standard solution, and *SC* represents the concentration of the sample (mg/mL). All the extracts were analyzed in triplicate.

### 2.3. β-Glucan and Other Glucan Content

A β-glucan assay kit for mushroom and yeast (K-YBGL, Megazyme, Wicklow, Ireland) was used for glucan content analysis in the roasted shiitake mushroom samples. Analysis of the total glucan, α-, and β-glucan was conducted simultaneously. For the total glucan analysis, 1.5 mL of 37% HCl (Sigma-Aldrich Chemical Company, St. Louis, USA) was added to 100 mg of a shiitake mushroom and then, placed in an ice water bath (0 °C) for 20 min. Then, 10 mL of distilled water was added and incubated at 100 °C for two hours. After the samples cooled down to room temperature, 10 mL of 2 N KOH (Sigma-Aldrich Chemical Company, St. Louis, MO, USA) was added to each sample and mixed thoroughly using a vortex mixer (VM-10, Daihan Scientific, Seoul, South) at a speed of 4000 rpm. Each sample was transferred to a volumetric flask and adjusted to 100 mL with a 0.2 M sodium acetate buffer at pH 5.0. The samples were then centrifuged at 10,000 rpm for 10 min. Then, 0.1 mL of supernatant was collected and 0.1 mL of exo-1, 3-β-glucanase (20 U/mL) and β-glucosidase (4 U/mL) solution was added to this supernatant and incubated at 40 °C for 60 min. Finally, 3 mL of glucose oxidase/peroxidase (GOPOD) solution was added to the final sample and were sit for 20 min at 40 °C to react. The total glucan was measured in the absorbance set at 510 nm using spectrophotometry (Biodrop Duo, Biodrop, Cambridge, United Kingdom) for each roasted shiitake mushroom sample.

Once the total glucan contents were analyzed, the α-glucan contents were analyzed as follows: 2 mL of 2 N KOH (Sigma-Aldrich Chemical Company, St. Louis, MI, USA) was added to 100 mg of a shiitake mushroom sample and placed in a 30 °C water bath for 45 min. Then, 8 mL of 1.2 M sodium acetate buffer (pH 3.8), 0.2 mL of amyloglucosidase (1630 U/mL), and an invertase (500 U/mL) solution were added. Then, the mixture was placed in water bath with a temperature set at 40 °C for 30 min. The samples were centrifuged at 4000 rpm for 10 min, and 0.1 mL of supernatant was transferred to a solution containing 0.1 mL of 0.2 M sodium acetate buffer (pH 5.0) and 3 mL of GOPOD. The final samples were reacted at 40 °C for 20 min. The α-glucan absorbance was measured at 510 nm using spectrophotometry. The glucose standard (1 mg/mL) solution was used as a control standard, and a 0.2 M sodium acetate buffer set at pH 5.0 was used for a blank. The determination of the β-glucan content was calculated by subtracting the α-glucan content from the total glucan content. Each content (%, *w/w*) was calculated using absorbance values with Mega-Calc™.

### 2.4. Volatile Flavor Analysis

The volatile flavor analysis was conducted by purge and trap (P&T) sampler (JTD-505III, Japan Analytical Industry, Tokyo, Japan) followed by gas chromatograph-mass spectrometry (GC-MS; QP2010 Plus, Shimadzu, Kyoto, Japan). Prior to the P&T sampling, roasted shiitake mushroom samples went through a bubbling process at a rate of 50 mL/min for 30 min at a temperature set at 60 °C in AQ-200 liquid sampler (Japan Analytical Industry, Tokyo, Japan) to capture the volatile aromatic compounds. Upon completion of bubbling process, volatile aromatic compounds were absorbed in Tenax GR (Japan Analytical Industry, Tokyo, Japan). This Tenax GR was transferred to the P&T sampler. The desorption temperature in the P&T sampler was set at 280 °C for 30 min at a rate of 50 mL/min, followed by the cold trap set at −40 °C, and pyrolysis was applied at the temperature set at 280 °C. The temperature for both transfer line and needle heater was also set at 280 °C. The head press for the P&T sampler was adjusted at 86 MPa, and flow rate in the column was set at 1.0 mL/min with a 1/100 split ratio.

For the quantification and qualification of volatile compounds, GC-MS QP2010 Plus equipped with DB-624 column (30 m × 0.251 mm × 1.40 mm; Agilent Technologies, Wilmington, DE, USA) was used. The temperature in the GC-MS oven was programmed as followings: 40 °C for 3-min hold, then the temperature was increased at a speed of 10 °C/minute up to 260 °C, then held for 5 min at 260 °C. The mass spectrometer was operated with full scan mode with positive electron impact ionization mode with 70 eV of electron energy. The scan range was set between 45 and 500 m/z. The peak area ratio (PAR) for each volatile compound was calculated as peak area height divided by total peak area. The volatile compounds were identified by mass spectrum in the Wiley mass spectral databases (NIST08, Wiley). All volatile flavor analysis was carried out at CURF in Jeonbuk National University.

### 2.5. Statistical Analysis

The antioxidant properties and glucan contents were expressed as the mean ± standard deviation of a triplicate analyses, and a significant difference among shiitake mushrooms roasted different temperatures was determined by analysis of variance (ANOVA) followed by Duncan’s multiple range test at the level of α = 0.05 for roasting-related attributes, antioxidant properties, peak area ratio, and α = 0.10 for glutan (α-, β-, and total) contents. Prior to run ANOVA, data normality and equal variance assumptions have been checked. A principal component analysis (PCA) was conducted to define the location of each shiitake mushroom sample in the antioxidant, glucan, and volatile flavor analysis map using 2018 XLSTAT software (Addinsoft, Paris, France). All other analyses were performed using SPSS (version 25.0; IBM, Amonk. New York, NY, USA).

## 3. Results

### 3.1. Antioxidant Properties

The length and weight of shiitake mushrooms before and after roasting according to the roasting temperature (80, 100, 120, 140, 160, 180 °C), and the corresponding reduction rate of length and weight, are shown in Table 1. It can be seen that the length reduction rate from 120 °C to 180 °C was 25% to 31%, significantly higher than the reduction rate at 80 °C and 100 °C (10.42, 14.01% respectively) (*p* < 0.05). Weight reduction rates also tended to be similar to length reduction rates. The lowest reduction rate (68.06%) at 80 °C was shown, and the length reduction rate was increased as the roasting temperature increased. It can be seen that the weight reduction rate at 140–180 °C was significantly higher than the reduction rate at the low roasting temperature (*p* < 0.05). Therefore, we could see that the rate of reduction in length and weight increased as the roasting temperature increased.

The antioxidant contents of shiitake mushrooms roasted at different temperatures are shown in Table 2. Significant differences between the shiitake samples were observed in the DPPH radical scavenging activity. The highest antioxidant contents were observed at 80 °C, 140 °C, and 160 °C with 83.74%, 82.32%, and 82.64%, respectively (*p* < 0.05). The highest free total phenol levels were observed in shiitake mushrooms roasted at 140 °C and 160 °C: 0.52 mg and 0.51 mg, respectively. The bound total phenol level was observed best at 140 °C. Therefore, the maximum total phenol content was seen at 140 °C. Like the total phenol content, the polyphenol content was expressed as mg of gallic acid compounds per 100 g of sample (weighed as is). The highest content of polyphenols was 0.81 mg at 140 °C and 160 °C, and the lowest content was observed as 0.62 mg at 80 °C and 180 °C. After heat treatment at 140 °C for 60 min, the TEAC values were 4.23 and 4.89 mg Trolox equivalents/100 g sample, respectively. Compared to previously reported antioxidant contents in shiitake mushrooms, the antioxidant contents reported in this study were in agreement with the antioxidant contents in other mushroom products. The highest antioxidant contents in shiitake mushrooms have been reported as 88.6% in shiitake mushrooms heated at 121 °C for 30 min. In addition, the free polyphenols were prone to increase as the temperature and the heating time increased [15]. An increase tendency of antioxidant properties with increased temperature was also reported [6]. Increase of antioxidant activities of shiitake mushroom with increase heat treatment temperatures may have been attributed from the deactivation of endogenous enzymes responsible for oxidation [15], and similar trend was also observed in many plant-origin products such as sweet corn [16], Citrus peel [17] and tomato [18].

### 3.2. β-Glucan, Total, and α-Glucan Analysis Results

The β-glucan and other glucan (total and α-) contents of shiitake mushrooms roasted at different temperatures are shown in Table 3. Significant differences in glucan were observed in the total α- and β-glucans among the shiitake mushroom samples (*p* < 0.10). The content of total glucan was at its highest when shiitake mushrooms were roasted at 120 °C for 60 min, in that 41.61% of total glucan was detected (*p* < 0.10), while the lowest total glucan levels were observed in shiitake mushrooms roasted at 160 °C (34.84%). The levels of α-glucan were generally lower than β-glucan, indicating that most of the glucan found in shiitake mushrooms is β-glucan, regardless of the roasting conditions. The highest content of β-glucan was shown at 120 °C, and the lowest at 160 °C (41.49% and 34.86%, respectively; *p* < 0.10), which is a similar pattern to that seen in total glucan.

The β-glucan contents reported in this study were in agreement with the β-glucan contents reported in other studies: One study reported the different levels of β-glucan in the cap and stalk of shiitake mushrooms prepared at 60 °C for 16 h as 19.78% and 35.31%, respectively [19]. Another study reported that the β-glucan content in shiitake mushrooms dried at 40 °C was 24.18% [20]. A β-glucan content of freeze-dried shiitake mushroom was reported around 28.3% of the total weight, which was similar to the quantity in heated shiitake mushrooms [21]. It is also worth noting that the total and β-glucan contents were proportional to the increase in temperature until 120 °C in this study. A decreasing tendency was observed at temperatures above 120 °C. Similar patterns have previously been reported, in that the release of β-glucan from barley increased as the extraction temperature increased [22]. Another study also reported an increase in β-glucan in shiitake mushrooms with an increase in the drying temperature of the mushrooms [23].

### 3.3. Volatile Flavor Analysis Results

**Figure 1:**
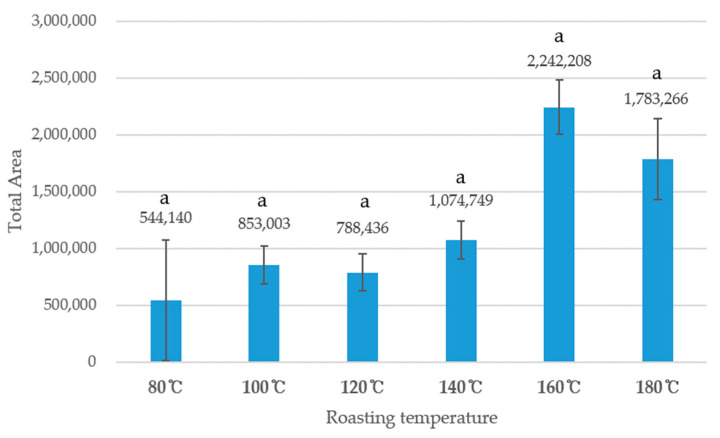
Total Peak Area of shiitake mushroom roasted in six different temperatures. 80 °C represents shiitake mushroom roasted at 80 °C; 100 °C represents shiitake mushroom roasted at 100 °C; 120 °C represents shiitake mushroom roasted at 120 °C; 140 °C represents shiitake mushroom roasted at 140 °C; 160 °C represents shiitake mushroom roasted at 160 °C. Alphabetical letter “a” followed by numbers represent no significant differences in peak area ration between shiitake mushroom samples roasted in different temperatures.

Figure 1 shows the total peak area of shiitake mushrooms roasted at six different temperatures. The shiitake mushrooms roasted at 160 °C had the highest total peak area with 2,242,208, followed by 180 °C (1,783,266), and 140 °C (1,074,749), indicating that the mushroom samples roasted at 160 °C had the most diverse volatile flavor profiles. The increment of the total peak area according to the increase in roasting temperature has been reported in a previous study [24].

Twenty-six volatile aromatic compounds were identified in the shiitake samples roasted at different temperatures (Table 4). The 26 volatile compounds identified included the following: eight aldehydes, including isobutyraldehyde, 3-methyl butanal, 2-methyl butanal, 2-methyl-2-butenal, pentanal, 2-methyl pentanal, hexanal, and benzaldehyde; four sulfur-containing compounds, namely carbon disulfide, thiophene, dimethyl disulfide, and 2-methylthiophene; four alcohols, including 2-pentanol, 3-methyl 1-butanol, 2-methyl 1-butanol, and 1-pentanol; three pyrazines, 2-methyl pyrazine, 2,5-dimethyl pyrazine, and 2,3,5-trimethyl pyrazine, and seven other compounds. Four compounds, namely benzene, 3-methyl butanal, 2-methyl butanal, and 1,3,5,7-cyclooctatetraene, were detected in all the shiitake mushroom samples roasted at six different temperatures.

As shown in Figure 1, the shiitake mushroom samples roasted at 160 °C had 21 different volatile compounds, while the other samples had between seven and 10 different volatile compounds. Previous studies have reported that alcohols are the major chemical family present in mature shiitake mushrooms, and the 1-octene-3-ol among alcohol group has been identified as the major volatile compound found in raw mature shiitake mushrooms [24,31]. In this study, 1-octene-3-ol was not found in the shiitake mushrooms, as all the samples were heated and dried. However, 2-methyl 1-butanol and 3-methyl-1butanol, which had whiskey, malty and burnt aromatics, were present in several shiitake mushroom samples (roasted at 80 °C, 100 °C, and 180 °C). In shiitake mushrooms roasted at 140 °C and 160 °C, 2-pentanol, which produces a balsamic aromatic was found, and 1-pentanol, which has a pungent aromatic, was found in mushrooms roasted at 160 °C. Regarding sulfur-containing compounds, carbon disulfide, thiophene, dimethyl disulfide, and 2-methylthiophene were identified in the shiitake mushroom samples. Straight-chain sulfur compounds, including dimethyl disulfide in this study, can provide onion and cabbage aromatics in general, which can produce the typical aromatics of fresh shiitake mushrooms and savory and meaty aromatics in dried mushrooms [10,24]. In this study, dimethyl disulfide was exclusively found in shiitake mushroom roasted in 160 °C with 2.26% PAR. It has been reported that the low boiling point of straight-chain sulfur-containing compounds can impact the decrease of concentration during the drying process [32]. Therefore, the absence of dimethyl disulfide and other straight-chain sulfur-containing compounds in shiitake mushroom samples may be attributed to the volatility of these compounds. Nutty and malty aromatic characteristics have previously been noted with 2-methyl butanal and 3-methyl butanel [11]. These two compounds were present in all the roasted shiitake mushroom samples in this study, ranging between 5.15% and 37.1% for 3-methyl butanal and 2% and 30.61% for 2-methyl butanal. These two compounds are Strecker aldehydes, formed by the Streker degradation process in various foods [11]. The presence of these two Strecker aldehydes may be attributed to the Strecker degradation process during the heated drying process in shiitake mushrooms.

### 3.4. Correlation of the Antioxidant Properties, Glucan Content, and Volatile Flavor Analysis Results

**Figure 2:**
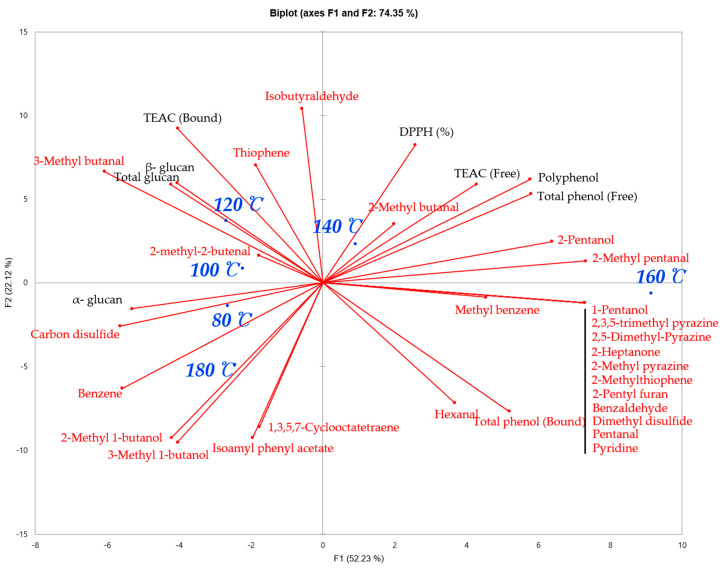
Principal Component Analysis Biplot on antioxidant, β-glucan and volatile flavor profiles of shiitake mushroom roasted in six different temperatures. 80 °C represents shiitake mushroom roasted at 80 °C; 100 °C represents shiitake mushroom roasted at 100 °C; 120 °C represents shiitake mushroom roasted at 120 °C; 140 °C represents shiitake mushroom roasted at 140 °C; 160 °C represents shiitake mushroom roasted at 160 °C.

Figure 2 shows a PCA biplot on the antioxidant, β-glucan, and volatile flavor profiles of shiitake mushrooms roasted at six different temperatures. This biplot demonstrates how each shiitake mushroom sample is located in the antioxidant, glucan contents (α-, β, and total) and volatile flavor profile map. The shiitake mushroom samples were differentiated by their volatile flavor profiles, in that the more abundant flavor compounds were present with an increase in roasting temperature. It is worth noting that the shiitake mushroom sample roasted at 180 °C shares similar volatile flavor profiles with mushrooms roasted at 80 °C, indicating a loss of volatile flavor compounds when roasting at too high a temperature. As for antioxidant properties, a high correlation between the total phenol (free), polyphenol contents, TEAC (free) and DPPH (%) were observed, and antioxidant properties were characteristic of shiitake mushrooms roasted at 140. The shiitake mushroom samples roasted at 100 °C and 120 °C had a high correlation to the total and β-glucan contents. These samples, high in glucan (total and β-), tended to have 2-methyl 2- butenal, 3-methyl butanal, and thiophene. The shiitake mushrooms roasted at 80 °C had a high correlation to α-glucan content and carbon disulfide. The correlation matrix of antioxidant activities, glucan contents, and instrumental flavor analysis results of shiitake mushrooms dried in 6 different degrees is illustrated in Table S1.

## 4. Conclusions

This study investigated the influence of roasting conditions on the volatile flavor profiles and functional properties of shiitake mushrooms. The results show that shiitake mushrooms roasted at 140 °C have high antioxidant activity. Shiitake mushrooms roasted at 160 °C have the most abundant flavor profiles. An increase in roasting temperature increases the overall flavor abundance up to 160 °C, while roasting at temperatures higher than 160 °C can decrease the overall flavor abundance of shiitake mushrooms. The finding from this study suggests the cooking direction of shiitake mushroom, in that roasting shiitake mushroom at 100–120 °C is recommended for functional benefits while roasting mushroom at higher temperature (160 °C) can maximize the authentic flavor of shiitake mushrooms.

## Figures and Tables

**Table 1:** Samples included in this study.

Roasting Conditions	Before Roasting		After Roasting			
	Length (cm)	Weight (g)	Length (cm)	Weight (g)	Length Reduction (%)	Weight Reduction (%)
80 °C 60 min	7.03 ± 0.05	2.63 ± 0.61	6.3 ± 0.24	0.84 ± 0.11	10.42 ± 3.55 b	68.06 ± 7.95 b
100 °C 60 min	7.6 ± 0.08	3.27 ± 0.46	6.53 ± 0.21	0.79 ± 0.11	14.01 ± 3.16 b	75.84 ± 6.80 ab
120 °C 60 min	5.87 ± 0.21	2.76 ± 0.45	4.03 ± 0.05	0.65 ± 0.10	31.14 ± 3.21 a	76.36 ± 2.37 ab
140 °C 60 min	6.83 ± 2.26	2.78 ± 0.33	4.73 ± 0.21	0.47 ± 0.03	30.75 ± 2.31 a	83.23 ± 1.26 a
160 °C 60 min	6.8 ± 0.41	2.95 ± 0.47	5.07 ± 0.41	0.54 ± 0.14	25.75 ± 5.47 a	81.83 ± 7.45 a
180 °C 60 min	7.1 ± 0.14	3.06 ± 0.63	5.27 ± 0.33	0.56 ± 0.09	25.87 ± 3.60 a	81.68 ± 0.88 a

Means in a column that does not share the same alphabetical letter represent significant difference at *p* < 0.05.

**Table 2:** Antioxidant properties of Shiitake mushroom roasted in different conditions.

	DPPH (%)	Total Phenol		Polyphenol	TEAC (mmol/L)	
		Free	Bound		Free	Bound
80 °C	83.74 ± 0.77 a	0.38 ± 0.02 c	1.28 ± 0.02 c	0.62 ± 0.03 c	2.13 ± 0.02 f	4.03 ± 0.01 d
100 °C	76.29 ± 1.24 c	0.42 ± 0.02 b	1.09 ± 0.02 d	0.70 ± 0.04 b	2.80 ± 0.01 e	4.60 ± 0.04 c
120 °C	78.87 ± 1.43 b	0.43 ± 0.00 b	0.98 ± 0.02 e	0.70 ± 0.01 b	3.62 ± 0.01 c	4.78 ± 0.01 b
140 °C	82.32 ± 0.37 a	0.52 ± 0.02 a	1.31 ± 0.03 c	0.81 ± 0.02 a	4.23 ± 0.00 a	4.89 ± 0.01 a
160 °C	82.64 ± 0.52 a	0.51 ± 0.02 a	1.54 ± 0.02 a	0.81 ± 0.02 a	3.86 ± 0.01 b	3.15 ± 0.02 f
180 °C	50.66 ± 1.51 d	0.39 ± 0.02 c	1.42 ± 0.03 b	0.62 ± 0.02 c	2.93 ± 0.00 d	3.43 ± 0.01 e

Means in a column that does not share the same alphabetical letter represent significant difference at *p* < 0.05.

**Table 3:** Total-, α- and β-glucan contents of shiitake mushroom roasted in different temperatures.

	Total Glucan (% *w/w*)	α-glucan (% *w/w*)	β-glucan (% *w/w*)
80 °C	36.93 ^a,b^ ± 2.89	0.24 ^a^ ± 0.15	36.69 ^a,b^ ± 2.88
100 °C	36.03 ^a,b^ ± 4.20	0.11 ^a^ ± 0.25	35.92 ^a,b^ ± 4.41
120 °C	41.61 ^a^ ± 3.95	0.12 ^a^ ± 0.09	41.49 ^a^ ± 4.02
140 °C	37.02 ^a,b^ ± 3.82	0.06 ^a^ ± 0.13	36.96 ^a,b^ ± 3.80
160 °C	34.84 ^b^ ± 4.08	0.02 ^a^ ± 0.21	34.86 ^b^ ± 4.20
180 °C	37.05 ^a,b^ ± 3.94	0.10 ^a^ ± 0.12	36.96 ^a,b^ ± 3.82

^a–c^ Geometric means within a column with different letters are different (*p* < 0.10).

**Table 4:** Volatile flavor analysis results of shitake mushroom roasted in different temperatures.

Peak #	R. Time				Peak Area Ratio (%)					
		Compound Name	Aroma Description	Ref.	80 °C	100 °C	120 °C	140 °C	160 °C	180 °C
1	4.013	Carbon disulfide	Solventy, sweet	[25]	3.68	6.06	3.1			4.05
2	4.927	Isobutyraldehyde	Malty, green, pungent	[26]	4.64	5.88	5.8	5.07	3.57	
3	7.182	Benzene	Sweet, solventy	[25]	23.27	11.83	17.8	11.38	6.46	26.54
4	7.359	3-methyl butanal	Nutty, malty	[11]	23.97	33.41	37.1	23.81	5.15	17.81
5	7.463	Thiophene	Garlic	[25]			30		1.24	
6	7.556	2-methyl butanal	Nutty, malty	[11]	11.39	23.4	6.2	30.61	16.52	9.34
7	7.659	2-methyl-2-butenal	Green, Fruit	[27]		2.75				
8	8.227	2-pentanol	Pungent	[28]				5.84	5.95	
9	8.438	Pentanal	Nutty, Malty	[29]					2.29	
10	9.484	Dimethyl disulfide	Onion, Cabbage	[9,24]					2.26	
11	9.67	3-methyl 1-butanol	Whiskey, malty, burnt	[26]	6.27	2.98				7.01
12	9.676	2-Methyl pentanal	-					4.25	7.22	
13	9.749	2-methyl 1-butanol	Malty, green, wine	[29]	4.89	3.53				6.01
14	9.742	Pyridine	-						6.21	
15	9.857	Methyl benzene	Paint	[30]	4.58			2.83	4.51	
16	9.873	Isoamyl phenyl acetate	-							6.25
17	10.036	2-Methylthiophene	Sulfur	[30]					1.64	
18	10.425	1-Pentanol	Pungent						2.17	
19	10.941	Hexanal	Grass, tallow, fat	[26]		2.2		3.96	5.76	7.19
20	11.495	2-methyl pyrazine	Popcorn	[30]					1.44	
21	12.9	1,3,5,7-Cyclooctatetraene	-		11.51	7.97	3.73	12.26	6.86	15.79
22	13.049	2-Heptanone	Soap	[26]					1.13	
23	13.366	2,5-dimethyl-Pyrazine	Peanut butter, solvent	[30]					1.44	
24	14.623	2-Pentyl furan	Beany	[29]					7.58	
25	14.987	Benzaldehyde	Almond, burnt sugar	[27]					2.43	
26	15.18	2,3,5-trimethyl pyrazine	-						2.03	

Peak area ratio (PAR) is the ratio of each peak area divided by total area.

## Data Availability

Data is contained within the article.

## Supplementary Materials

The following are available online at https://www.mdpi.com/2304-8158/10/1/54/s1, Table S1: Correlation matrix among instrumental flavor analysis, antioxidant activities, and functional activity (glucan contents) results.
