# Supplementary material for: Influence of Roasting Temperatures on the Antioxidant Properties, β-Glucan Content, and Volatile Flavor Profiles of Shiitake Mushroom
Source: Foods. 2020 Dec 28;10(1):54. doi: 10.3390/foods10010054 (PMC7824100; doi:10.3390/foods10010054)
Supplement: Supplementary file 1 [file foods-10-00054-s001.pdf]

Supplementary table 1. Correlation matrix among instrumental flavor analysis, antioxidant acti

Variables	Carbon disulfide	Isobutyraldehyde	Benzene	3-methyl butanal
Carbon disulfide	**1**	0.016	**0.500**	**0.586**
Isobutyraldehyde		**1**	**-0.508**	**0.590**
Benzene			**1**	**0.228**
3-methyl butanal				**1**
Thiophene				
2-methyl butanal				
2-methyl-2-butenal				
2-pentanol				
Pentanal				
Dimethyl disulfide				
3-methyl 1-butanol				
2-Methyl pentanal				
2-methyl 1-butanol				
Pyridine				
Methyl benzene				
Isoamyl phenyl acetate				
2-Methylthiophene				
1-Pentanol				
Hexanal				
2-methyl pyrazine				
1,3,5,7-Cyclooctatetraene				
2-Heptanone				
2,5-dimethyl-Pyrazine				
2-Pentyl furan				
Benzaldehyde				
2,3,5-trimethyl pyrazine				
DPPH				
Total phenol (Free)				
Total phenol (Bound)				
Polyphenol				
TEAC (Free)				
TEAC (Bound)				
Total glucan				
α- glucan				
β- glucan				

α=0.05

bold text=significant

ivities, and functional activity (glucan contents) results

Thiophene	2-methyl butanal	2-methyl-2-butenal	2-pentanol	Pentanal	Dimethyl disulfide
**0.035**	**-0.302**	**0.663**	**-0.910**	**-0.575**	**-0.575**
**0.361**	**0.343**	**0.382**	**0.054**	**-0.131**	**-0.131**
**0.076**	**-0.630**	**-0.279**	**-0.736**	**-0.620**	**-0.620**
**0.553**	**-0.055**	**0.423**	**-0.620**	**-0.789**	**-0.789**
**1**	**-0.534**	**-0.210**	**-0.292**	**-0.160**	**-0.160**
	**1**	**0.378**	**0.606**	0.015	0.015
		**1**	**-0.316**	**-0.200**	**-0.200**
			**1**	**0.641**	**0.641**
				**1**	**1.000**
					**1**

3-methyl 1-butanol	2-Methyl pentanal	2-methyl 1-butanol	Pyridine	Methyl benzene	Isoamyl phenyl acetate
**0.607**	**-0.867**	**0.701**	**-0.575**	**-0.615**	**0.252**
**-0.584**	-0.011	**-0.547**	**-0.131**	**0.085**	**-0.923**
**0.824**	**-0.760**	**0.766**	**-0.620**	**-0.291**	**0.657**
**-0.043**	**-0.742**	0.016	**-0.789**	**-0.620**	**-0.246**
**-0.427**	**-0.269**	**-0.450**	**-0.160**	**-0.410**	**-0.210**
**-0.380**	**0.438**	**-0.314**	0.015	**0.187**	**-0.365**
**0.041**	**-0.301**	**0.200**	**-0.200**	**-0.430**	**-0.200**
**-0.643**	**0.957**	**-0.678**	**0.641**	**0.578**	**-0.316**
**-0.407**	**0.837**	**-0.429**	**1.000**	**0.546**	**-0.200**
**-0.407**	**0.837**	**-0.429**	**1.000**	**0.546**	**-0.200**
**1**	**-0.613**	**0.985**	**-0.407**	**-0.097**	**0.645**
	**1**	**-0.646**	**0.837**	**0.620**	**-0.301**
		**1**	**-0.429**	**-0.201**	**0.642**
			**1**	**0.546**	**-0.200**
				**1**	**-0.430**
					**1**

2-Methyl thiophene	1-Pentanol	Hexanal	2-methyl pyrazine	1,3,5,7-Cyclooctatetraene	2-Heptanone
**-0.575**	**-0.575**	**-0.317**	**-0.575**	**0.049**	**-0.575**
**-0.131**	**-0.131**	**-0.787**	**-0.131**	**-0.696**	**-0.131**
**-0.620**	**-0.620**	**-0.093**	**-0.620**	**0.535**	**-0.620**
**-0.789**	**-0.789**	**-0.730**	**-0.789**	**-0.321**	**-0.789**
**-0.160**	**-0.160**	**-0.509**	**-0.160**	**-0.693**	**-0.160**
0.015	0.015	**0.178**	0.015	**0.163**	0.015
**-0.200**	**-0.200**	**-0.162**	**-0.200**	**-0.194**	**-0.200**
**0.641**	**0.641**	**0.437**	**0.641**	-0.027	**0.641**
**1.000**	**1.000**	**0.423**	**1.000**	**-0.320**	**1.000**
**1.000**	**1.000**	**0.423**	**1.000**	**-0.320**	**1.000**
**-0.407**	**-0.407**	**0.106**	**-0.407**	**0.695**	**-0.407**
**0.837**	**0.837**	**0.472**	**0.837**	**-0.141**	**0.837**
**-0.429**	**-0.429**	**0.122**	**-0.429**	**0.665**	**-0.429**
**1.000**	**1.000**	**0.423**	**1.000**	**-0.320**	**1.000**
**0.546**	**0.546**	-0.023	**0.546**	**0.059**	**0.546**
**-0.200**	**-0.200**	**0.657**	**-0.200**	**0.691**	**-0.200**
**1**	**1.000**	**0.423**	**1.000**	**-0.320**	**1.000**
	**1**	**0.423**	**1.000**	**-0.320**	**1.000**
		**1**	**0.423**	**0.527**	**0.423**
			**1**	**-0.320**	**1.000**
				**1**	**-0.320**
					**1**

2,5-dimethyl-Pyrazine	2-Pentyl furan	Benzaldehyde	2,3,5-trimethyl pyrazine	DPPH	Total phenol (Free)
**-0.575**	**-0.575**	**-0.575**	**-0.575**	**-0.393**	**-0.842**
**-0.131**	**-0.131**	**-0.131**	**-0.131**	**0.835**	**0.242**
**-0.620**	**-0.620**	**-0.620**	**-0.620**	**-0.616**	**-0.841**
**-0.789**	**-0.789**	**-0.789**	**-0.789**	**0.097**	**-0.392**
**-0.160**	**-0.160**	**-0.160**	**-0.160**	**0.133**	**-0.073**
0.015	0.015	0.015	0.015	**0.348**	**0.636**
**-0.200**	**-0.200**	**-0.200**	**-0.200**	0.021	**-0.177**
**0.641**	**0.641**	**0.641**	**0.641**	**0.414**	**0.949**
**1.000**	**1.000**	**1.000**	**1.000**	**0.268**	**0.560**
**1.000**	**1.000**	**1.000**	**1.000**	**0.268**	**0.560**
**-0.407**	**-0.407**	**-0.407**	**-0.407**	**-0.604**	**-0.833**
**0.837**	**0.837**	**0.837**	**0.837**	**0.397**	**0.889**
**-0.429**	**-0.429**	**-0.429**	**-0.429**	**-0.634**	**-0.838**
**1.000**	**1.000**	**1.000**	**1.000**	**0.268**	**0.560**
**0.546**	**0.546**	**0.546**	**0.546**	**0.604**	**0.365**
**-0.200**	**-0.200**	**-0.200**	**-0.200**	**-0.976**	**-0.423**
**1.000**	**1.000**	**1.000**	**1.000**	**0.268**	**0.560**
**1.000**	**1.000**	**1.000**	**1.000**	**0.268**	**0.560**
**0.423**	**0.423**	**0.423**	**0.423**	**-0.604**	**0.319**
**1.000**	**1.000**	**1.000**	**1.000**	**0.268**	**0.560**
**-0.320**	**-0.320**	**-0.320**	**-0.320**	**-0.589**	**-0.242**
**1.000**	**1.000**	**1.000**	**1.000**	**0.268**	**0.560**
**1**	**1.000**	**1.000**	**1.000**	**0.268**	**0.560**
	**1**	**1.000**	**1.000**	**0.268**	**0.560**
		**1**	**1.000**	**0.268**	**0.560**
			**1**	**0.268**	**0.560**
				**1**	**0.462**
					**1**

Total phenol (Bound)	Polyphenol	TEAC (Free)	TEAC (Bound)	Total glucan	α- glucan
**-0.543**	**-0.770**	**-0.771**	**0.157**	**0.152**	**0.557**
**-0.670**	**0.350**	**0.135**	**0.744**	**0.273**	**0.156**
**-0.110**	**-0.911**	**-0.645**	**-0.109**	**0.367**	**0.680**
**-0.976**	**-0.322**	**-0.203**	**0.852**	**0.702**	**0.445**
**-0.666**	**-0.034**	**0.243**	**0.399**	**0.914**	**0.053**
**0.124**	**0.645**	**0.415**	**0.377**	**-0.502**	**-0.407**
**-0.427**	**-0.057**	**-0.291**	**0.303**	**-0.259**	0.011
**0.585**	**0.908**	**0.778**	**-0.143**	**-0.446**	**-0.713**
**0.640**	**0.574**	**0.377**	**-0.667**	**-0.512**	**-0.582**
**0.640**	**0.574**	**0.377**	**-0.667**	**-0.512**	**-0.582**
**0.171**	**-0.880**	**-0.850**	**-0.369**	**-0.186**	**0.636**
**0.660**	**0.865**	**0.698**	**-0.355**	**-0.513**	**-0.729**
**0.111**	**-0.867**	**-0.856**	**-0.326**	**-0.222**	**0.586**
**0.640**	**0.574**	**0.377**	**-0.667**	**-0.512**	**-0.582**
**0.588**	**0.333**	0.029	**-0.353**	**-0.497**	**0.081**
**0.356**	**-0.517**	**-0.209**	**-0.480**	**-0.042**	**-0.055**
**0.640**	**0.574**	**0.377**	**-0.667**	**-0.512**	**-0.582**
**0.640**	**0.574**	**0.377**	**-0.667**	**-0.512**	**-0.582**
**0.777**	**0.238**	**0.334**	**-0.636**	**-0.549**	**-0.681**
**0.640**	**0.574**	**0.377**	**-0.667**	**-0.512**	**-0.582**
**0.517**	**-0.359**	**-0.275**	**-0.278**	**-0.391**	**0.150**
**0.640**	**0.574**	**0.377**	**-0.667**	**-0.512**	**-0.582**
**0.640**	**0.574**	**0.377**	**-0.667**	**-0.512**	**-0.582**
**0.640**	**0.574**	**0.377**	**-0.667**	**-0.512**	**-0.582**
**0.640**	**0.574**	**0.377**	**-0.667**	**-0.512**	**-0.582**
**0.640**	**0.574**	**0.377**	**-0.667**	**-0.512**	**-0.582**
**-0.197**	**0.530**	**0.217**	**0.375**	-0.019	**0.079**
**0.332**	**0.988**	**0.900**	**0.078**	**-0.257**	**-0.795**
**1**	**0.242**	**0.147**	**-0.811**	**-0.730**	**-0.382**
	**1**	**0.872**	**0.124**	**-0.253**	**-0.784**
		**1**	**0.179**	**0.099**	**-0.838**
			**1**	**0.548**	**0.187**
				**1**	**0.271**
					**1**

β- glucan
**0.132**
**0.269**
**0.346**
**0.690**
**0.922**
**-0.495**
**-0.264**
**-0.424**
**-0.492**
**-0.492**
**-0.211**
**-0.490**
**-0.246**
**-0.492**
**-0.502**
**-0.040**
**-0.492**
**-0.492**
**-0.529**
**-0.492**
**-0.401**
**-0.492**
**-0.492**
**-0.492**
**-0.492**
**-0.492**
-0.021
**-0.231**
**-0.721**
**-0.227**
**0.130**
**0.543**
**0.999**
**0.238**
**1**
